# Description of the technique for laparoscopic radical prostatectomy in canine cadavers: 2D vs. 3D camera

**DOI:** 10.1371/journal.pone.0274868

**Published:** 2022-11-29

**Authors:** Eric Monnet, Ahmed Hafez

**Affiliations:** 1 College of Veterinary Medicine, Dept of Clinical Sciences, Colorado State University, Fort Collins, CO, United States of America; 2 Faculty of Veterinary Medicine, Beni-Suef University, Beni Suef, Egypt; AIIMS: All India Institute of Medical Sciences, INDIA

## Abstract

**Objective:**

To describe the technique of laparoscopic radical prostatectomy in canine cadavers.

**Study design:**

Cadaveric study.

**Animals:**

8 canine cadavers.

**Methods:**

Specimens were randomly divided into a 2D or 3D group. The vesicourethral anastomosis (VUA) was performed with 5 mm laparoscopic needle holders. A unidirectional barbed suture was used to complete the VUA with two simple continuous suture patterns. The number of stitches placed, the patency of the anastomosis, and the distance between the VUA and the ureters were recorded.

**Results:**

Four dogs were entered into each group. The prostatectomy was completed in each dog following the same technique. VUA were completed with nine stitches (range: 8–10 stitches) for the 2D group and ten stitches (range: 9–11 stitches) for the 3D group (p<0.176). All the stitches were placed full thickness. The VUA was patent in each case. The left ureter was 1.05 cm (range: 0.5 to 1.1cm) from the VUA in the 2D group and 1.8 cm (range: 1.3–1.8 cm) for the 3D group (p< 0.025). The right ureter was 1.5 cm (range: 1 to 2 cm) from the VUA in the 2D group and 1.75 cm (range: 1.3–2 cm) for the 3D group (p< 0.55).

**Conclusion:**

Laparoscopic radical prostatectomy can be performed with a 2D or a 3D camera in canine cadavers. The 3D camera results in more accurate placement of the sutures since they were placed further away from the left ureter.

**Clinical significance:**

Radical prostatectomy with laparoscopy should be considered for dogs.

## Introduction

Radical prostatectomy (RP) is indicated mainly for resection of prostatic neoplasia [1–4]. High rates of complications have been reported in dogs following prostatectomy [3, 5, 6]. The most common complication is urinary incontinence making this surgery rarely performed.

Laparoscopic radical prostatectomy (LRP) in human patients has been associated with a significant reduction of major complications, especially urinary incontinence, because it allows sparing of the neurovascular pedicles on each side of the prostate [7]. Laparoscopy is beneficial and more practical for ablating small organs that are difficult to reach during the open approach. Robotic radical prostatectomy has been performed successfully in one client-owned dog and described in three canine cadavers [8, 9]. Robots are not readily available in veterinary surgery yet.

Vesicourethral anastomosis (VUA) is an essential component of RP. It requires an appropriate tissue apposition to minimize leakage in the postoperative period and to improve long-term outcomes [10–12]. The utilization of a 3D camera has been recommended for delicate dissection and suturing [13–15].

Our study objectives were to describe the technique for laparoscopic radical prostatectomy in canine cadavers and report using a 3D camera vs. a 2D camera for the laparoscopic radical prostatectomy. We hypothesized that using a 2D or 3D camera does not affect laparoscopic radical prostatectomy in canine cadavers.

## Materials and methods

Frozen intact male canine cadavers were purchased for the study (Skulls Unlimited, skullsunlimited.com) (Colorado State University IACUC No. 17-7102A).

Cadavers were randomly divided into two groups for surgery: the 2D group and the 3D group. In the 2D group, a 10 mm endoscope with a 2D camera (Image 1 S: Tipcam camera, Karl Storz, Goleta, Ca) was used, while a 10 mm endoscope with a 3D camera was used in the 3D group (Image 1 S: Tipcam camera, Karl Storz, Goleta, Ca). The camera could be switched from 2D to 3D mode. Glasses were used with the camera in 3D mode. The number of suture bites needed for the construction of VUA, patency of the VUA, and distance between the ureters and the VUA were recorded. A 6” 4–0 Vloc 90 (Vloc, Medtronic, Minneapolis, MN) suture was used with a CV 23 17 mm ½ circle taper needle. All the procedures were performed but the same board certified surgeon.

### Surgical technique

**Fig 1:**
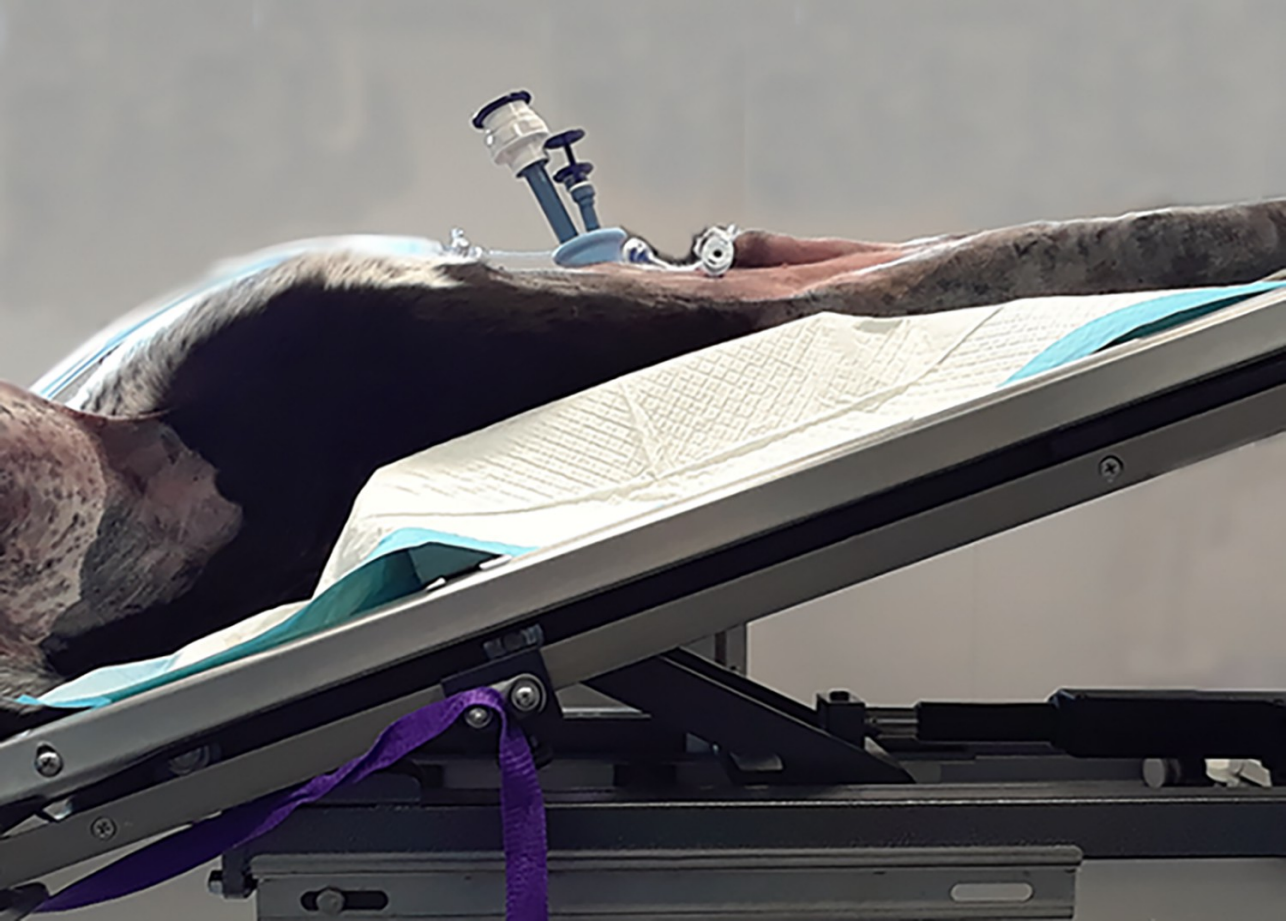
Canine cadaver in a severe Trendelenburg position for laparoscopic radical prostatectomy.

**Fig 2:**
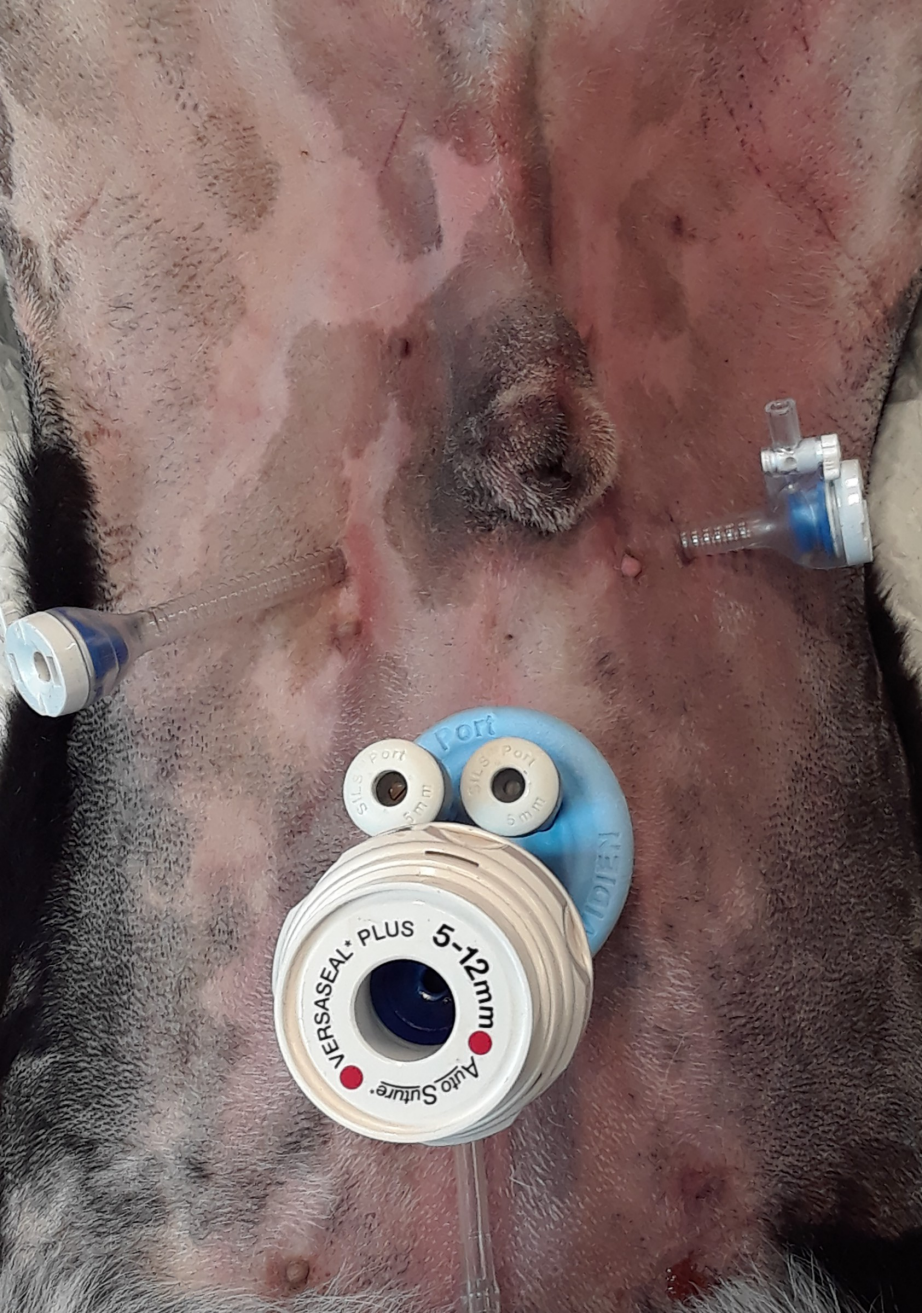
Outside view showing the placement of the single access port and the two 5 mm cannula on the left and right sides of the abdominal cavity.

The surgical technique was designed following the technique described in human patients [16, 17]. Dogs were placed in dorsal recumbency in a severe Trendelenburg position to help remove organs in the caudal abdomen (Fig 1). A single access port and two 5 mm cannulas were used. The single access port with two 5 mm cannulas and one 5–12 mm cannula for the 10 mm endoscope/camera was placed caudal to the umbilicus. One cannula was placed in the left and right side of the abdominal cavity at the level of the tip of the prepuce 3 to 4 cm lateral to midline (Fig 2). The abdominal cavity was insufflated to 12 mm Hg with carbon dioxide.

**Fig 3:**
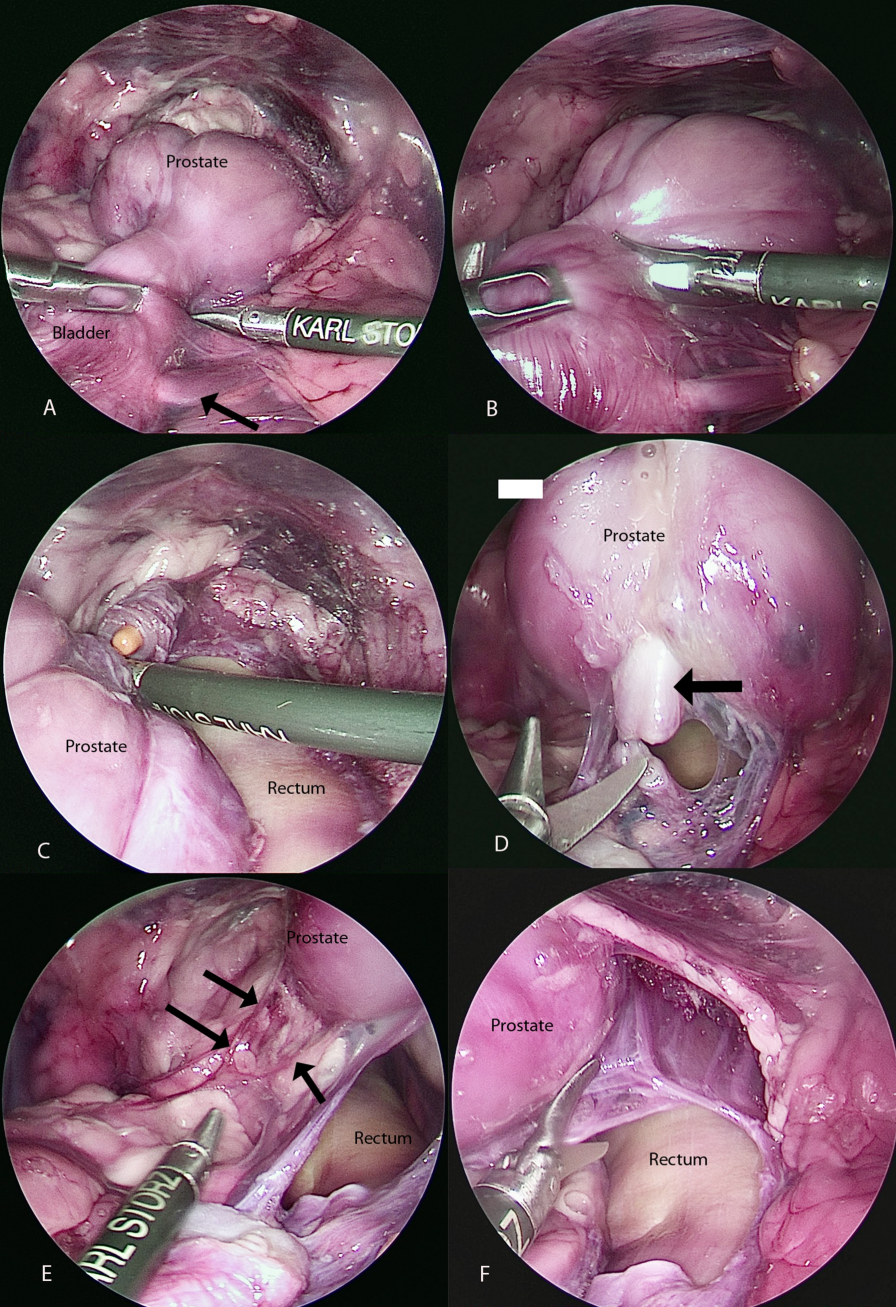
**A:** Dissection on the right side of the prostate. The right ureter is visible (black arrow). **B:** The urethra is transected between the prostate and the bladder from right to left with Metzembaum scissors. **C:** The urethra is almost completely transected, and the urinary catheter is visible. **D:** The ductus deferents have been transected with scissors. **E:** The left neurovascular pedicle is visible on each side of the prostate (black arrow). **F:** The dissection between the rectum and the prostate is completed.

The ventral ligament of the bladder was dissected first from the midline with a 5 mm Metzemabum scissor (Metzemabum scissors, Karl Storz, Goleta, Ca). A 5 mm grasping forceps (Fine Teeth Babcock, Karl Storz, Goleta, Ca) was used to retract the bladder cranially and dorsally. The peritoneum was then dissected from the pubic rim to enter the space of Retzius. The prostate was exposed. Dissection was conducted along with the capsule of the prostate and the trigone starting on the right side and extending dorsally (Fig 3A). The dissection was extended from the left side to connect with the previous dorsal plane of dissection.

**Fig 4:**
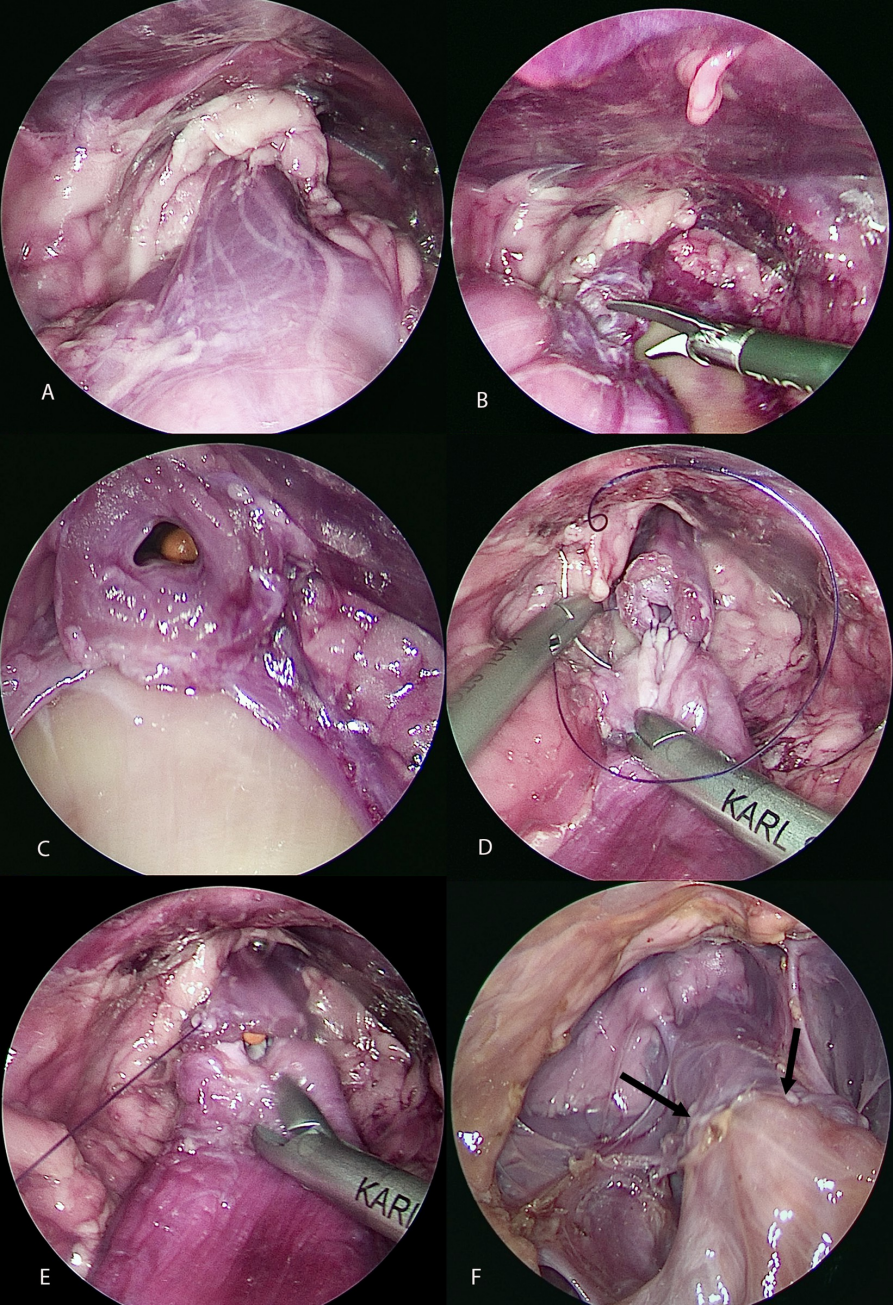
**A:** The urethra caudal to the prostate is now exposed. **B:** The urethra is transected caudal to the prostate. **C:** The urethra is now completely transected, and the urinary catheter is visible. **D:** The VUA is performed on the dorsal side with a continuous pattern of 4–0 V Loc 90 suture. The suture is placed outside-in in the bladder and inside-out in the urethra. **E:** The dorsal line of the suture has been completed, and the ventral side is started. **F:** The VUA is completed (black arrows).

The urethra was transected with 5 mm Metzenbaum scissors (Metzenbaum Scissors, Karl Storz, Goleta, Ca) between the cranial pole of the prostate and the trigone as close as possible to the prostate without entering the capsule (Fig 3B and 3C). The urine present in the bladder was aspirated from the abdominal cavity and the bladder. The prostate was elevated in the pelvic canal to visualize the ductus deferences. They were transected with 5 mm Metzembaum scissors (Metzemabum scissors, Karl Storz, Goleta, Ca) (Fig 3D). The neurovascular pedicle on the left and right sides of the prostate were dissected with Metzembaum scissors, avoiding tension on the pedicle (Fig 3E). The prostate was then dissected from the rectum after cranioventral retraction of the prostate (Fig 3F). After complete prostate dissection, the urethra was transected caudal to the prostate without entering the capsule (Fig 4A and 4B).

The bladder and the urethra were approximated together with grasping forceps. An 8 Fr urinary catheter was advanced in the urethra to the level of the transection (Fig 4C). The vesicourethral anastomosis was performed with 5 mm laparoscopic needle holders (Needle holders, Karl Storz, Goleta, Ca). Two 8” 4–0 glycomer 631 (Bison, Medtronic, Minneapolis, Mn) sutures with a CV 23 needle were used to complete the VUA. The sutures were placed from outside-in the bladder and inside-out in the urethra. The simple continuous suture patterns were started laterally at 3 o’clock. The dorsal side of the VUA was completed first (Fig 4D and 4E). Then the urinary catheter was advanced in the bladder, and the ventral side of the VUA was completed (Fig 4F). The two strands of the suture met together at 9 o’clock.

**Fig 5:**
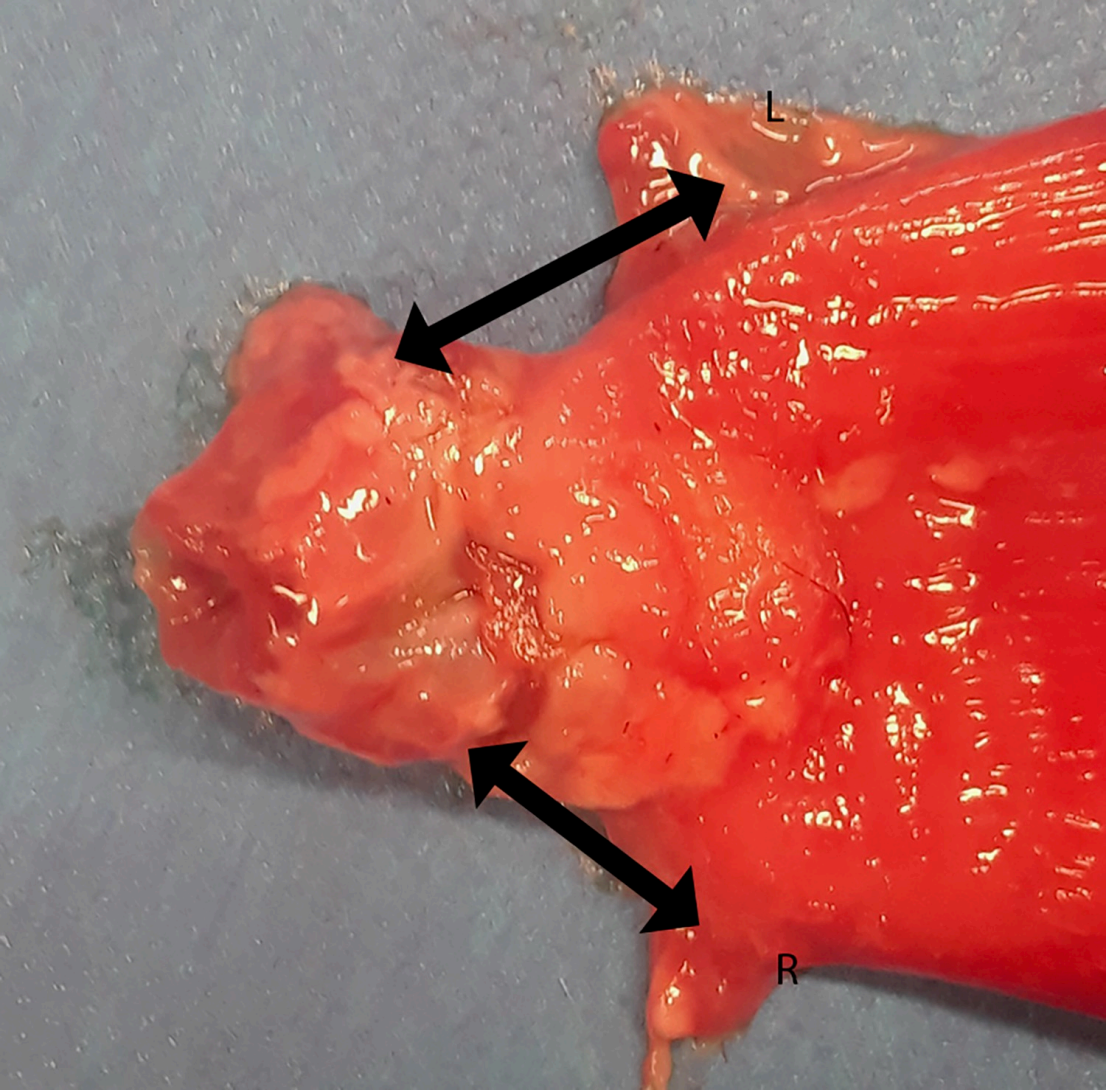
Bladder and urethra removed from the abdominal cavity with the left (L) and right (R) ureters. The distance between each ureter and the VUA was measured (black arrow).

**Fig 6:**
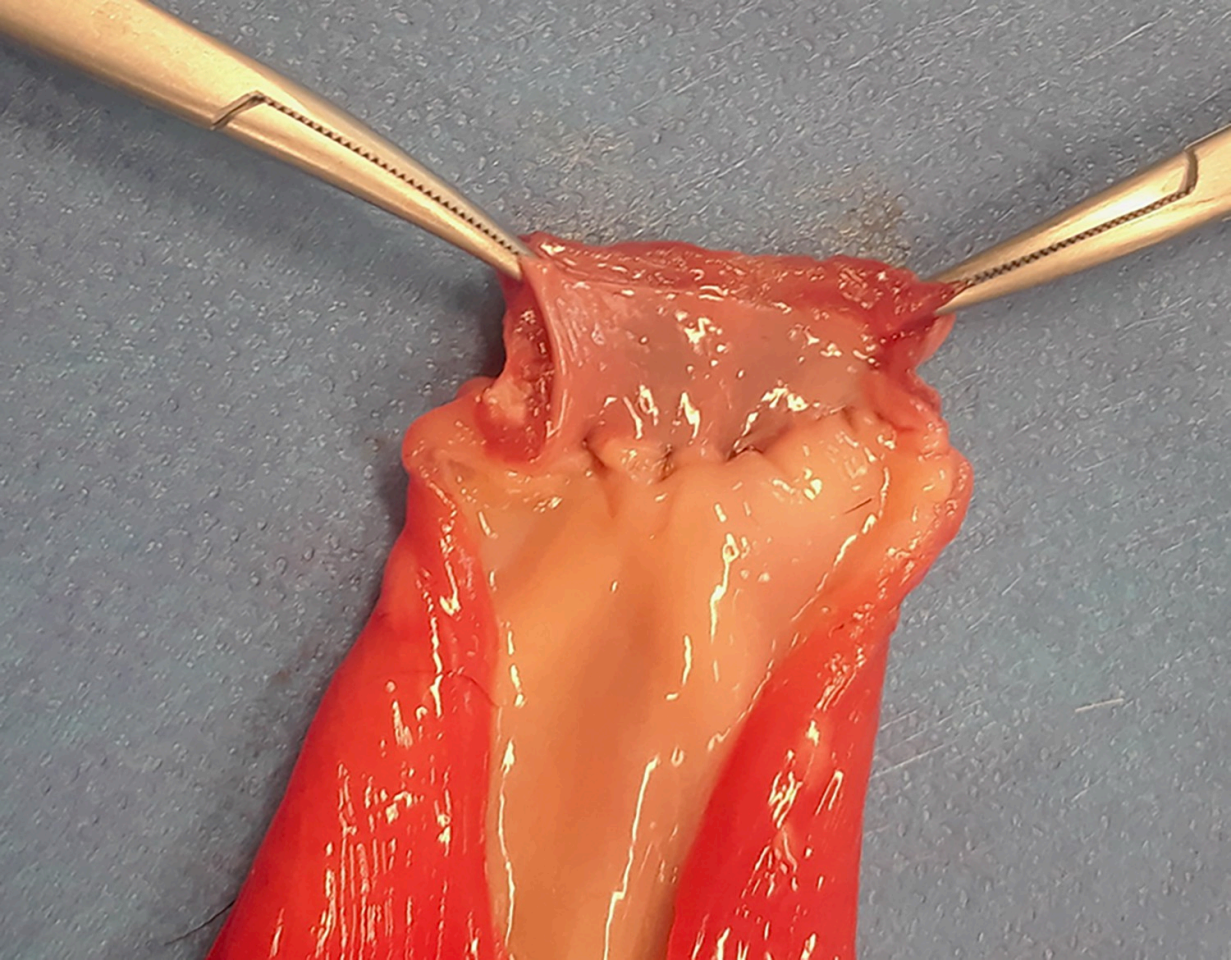
Urethra and bladder have been incised along the long axis to observe the suture line and the apposition of the mucosa. This VUA was graded as a 2.

The abdomen was open after completing the VUA, and the bladder/urethra specimen was collected. A 10 Fr urinary catheter was advanced through the VUA to evaluate patency. The distance from each ureter to the VUA was measured with a caliper (Fig 5). The urethra and bladder were then incised along their long axis, and the anastomosis was macroscopically evaluated. The spacing of the stitches was assessed by two observers not blinded to the group (Fig 6). The spacing was graded from 1 to 3, with one being attributed to an even spacing of the stitches with a 2mm gap between stitches. A spacing grade of 2 was attributed to even spacing but larger than 2 mm (Fig 4) and 3 for uneven spacing.

### Statistical analysis of the data

The number of stitches needed to make the constructs and the distance between the ureters and the VUA were reported as median and range. Wilcoxon ranks test was used to compare these variables between the 2D and 3D groups. A Chi-square was used to compare the distribution of the spacing grade between the 2D and 3D groups. Statistical testing was done with the commercially available statistical program (JMP, 13.2, SAS, Inc., 2016, Cary, NC). The level of significance was set at *P* < .05.

## Results

**Fig 7:**
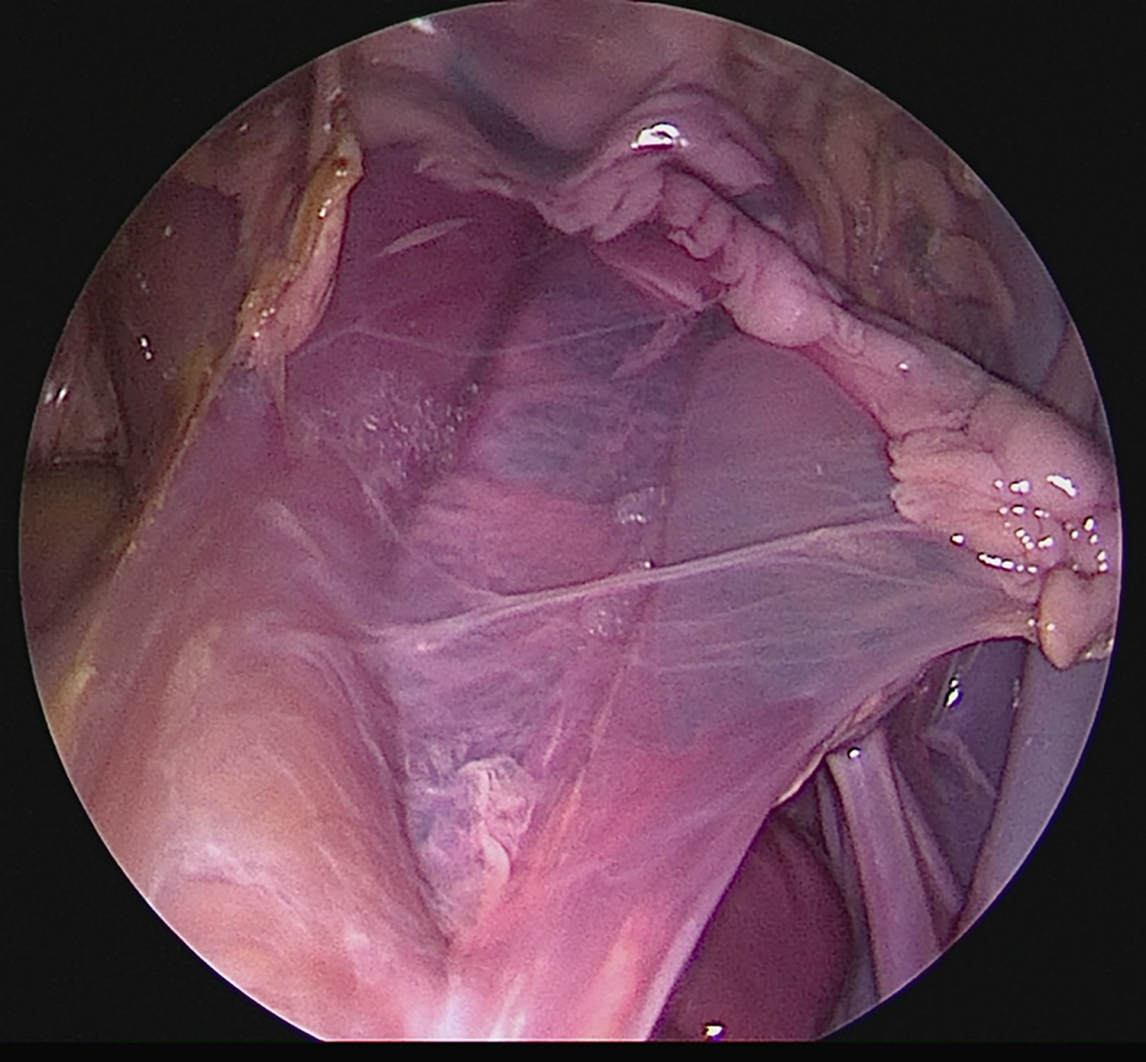
Visualization of the neurovascular bundle on the right side with exposure of the branching in the capsule of the prostate.

Four intact male canine cadavers were randomly entered into each group. The median weight was 27.5 Kg (range 20–30 Kg) for the dogs in the 2 D group and 30 Kg (range: 25–30 kg) for the dogs in the 3 D group. The prostate appeared normal-sized in each dog. The prostatectomy was performed successfully in each case with appropriate visualization of the neurovascular pedicles (Figs 3E and 7). VUA was completed in all cases in both groups. All constructs were patent, and no urethral narrowing was observed in any sample after completion of the anastomosis as the 10 Fr catheter could easily pass through the anastomotic site in both groups. No evidence of tissue tearing, or suture breakage was noticed on visual inspection of the VUA in both groups.

The VUA was completed with nine stitches (range: 8–10 stitches) for the 2D group and ten stitches (range 9–11 stitches) for the 3 D group (p<0.176). All the stitches were placed full thickness. The left ureter was 1.05 cm (range: 0.5 to 1.1cm) from the VUA in the 2D group and 1.8 cm (range: 1.3–1.8 cm) for the 3D group (p< 0.025). The right ureter was 1.5 cm (range: 1 to 2 cm) from the VUA in the 2D group and 1.75 cm (range: 1.3–2 cm) for the 3D group (p< 0.55). The distribution of the spacing grade for the dorsal and ventral suture line are reported in Table 1. The distribution was not significantly different between the 2D and the 3D camera for the dorsal (p = 0.170) and the ventral (p = 0.216) suture lines.

**Table 1:** Distribution of the spacing grades for the dorsal and the ventral suture lines performed with a 2-D and a 3-D camera.

	2D camera			3 D camera		
	Grade 1	Grade 2	Grade 3	Grade 1	Grade 2	Grade 3
**Dorsal line**	2	1	1	4	0	0
**Ventral Line**	3	1	0	4	0	0

## Discussion

Laparoscopic radical prostatectomy was completed with a 2D or a 3D camera in male canine cadavers, following the steps described for human radical prostatectomy. The technique to perform a radical prostatectomy has been described step by step. With the 3D camera, the left ureter was at a greater distance from the VUA, reducing the risk of iatrogenic injury to the left ureter.

Lovegrove et al. [17] designed a training protocol for human surgeons to learn robotic radical prostatectomy. This protocol was adapted to canine anatomy and laparoscopic surgery for our study. Since dogs do not have a dorsal venous plexus and seminal vesicles, those steps were not performed. The two most challenging parts were the dissection of the neurovascular pedicles and the VUA, as reported in the training protocol from Lovegrove et al. [17]. The utilization of a 3D camera subjectively facilitated the suturing for VUA because of better spatial orientation and depth perception. The accuracy of suturing during laparoscopic radical prostatectomy has significantly improved with a 3D camera [13].

The canine cadavers were placed in a severe Trendelenburg position to remove all the intestinal loops from the abdominal cavity’s caudal part. In live dogs, artificial ventilation will be required to maintain adequate ventilation because of the pressure placed on the diaphragm by the abdominal organs. With this position, the visualization of the bladder and the prostate was greatly facilitated. The cranial movement of the bladder did not seem to add tension on the suture line. An insufflation pressure of 12 mm Hg was used in this study. Lower pressure can be more likely used because the work is conducted in the pelvic canal. This would reduce the pressure on the diaphragm during the surgery.

Ureters must be identified during the entire procedure when the urethra is transected between the trigone and the cranial pole of the prostate and when the VUA is completed. Identification of the ureters is not described in the training protocol that Lovegrove et al. [17]. In the canine cadavers, the ureters were very close to our surgical field. With a grasper retracting the bladder in the appropriate direction, ureters were visible during the entire procedure reducing the risk of iatrogenic trauma during dissection and suturing. The distance between the left ureter and VUA was longer with the 3D camera than with the 2 D camera. This is more likely due to better depth perception with the 3D camera. However, on the right side, the distance between the right ureter and VUA was not significantly different but was longer with the 3D camera than with the 2D camera. The distance was affected mainly on the left side, more likely because the left side of the VUA was more challenging for a right-handed surgeon, and the 3D may have given us a better visualization. The distances from the ureter to the VUA were measured from the outside of the bladder and not the inside, where the ureters enter the mucosa of the bladder, because this is the only landmark we could see during surgery. The ureteral papillae were not visible during the completion of the VUA.

The urethra could be transected without entering the prostate capsule in normal canine cadavers. A right-handed surgeon performed the transection right to left with Metzenbaum scissors. It is paramount to keep a good orientation of the scissor during the transection to ensure the prostate’s capsule is not entered or the left ureter is not damaged. The 3D camera facilitated this step.

Both ductus deferents were visible dorsally after retraction of the prostate in a ventral direction. Endoclips would be required in a live patient to ligate the ductus deferents. There is no dorsal venous plexus in dogs that need to be controlled with ligature like in humans [16]. Also, dogs do not have seminal vesicles, which seems to facilitate the dissection of the prostate to reach the caudal part of it and visualize the caudal urethra.

Magnification and light allowed for good visualization of the neurovascular pedicles on either side of the prostate. The neuromuscular pedicles were equally visible with the 2D and the 3D cameras. It has been recommended in the human literature to dissect those neurovascular pedicles as close to the prostate as possible with cold dissection and minimal traction to minimize neuropraxia [16, 18]. Hemostasis was not required in this study since we used cadavers. Hemostasis should be performed with sutures or endoclips to reduce thermal injury to the nerves [16, 18]. Since there is no dorsal venous plexus like in humans, the dissection and the hemostasis of the neurovascular pedicle should be possible with endoclips. In humans, the dorsal venous plexus requires the placement of a suture to obtain adequate hemostasis [16].

The prostate shared a loose facia with the rectum dorsally. This facia must be gently dissected without entering the wall of the rectum. In human rhabomyosphincter being part of the Denonvillier’s facia posterior to the bladder has been described [16, 19]. It is used to reconstruct the VUA [16, 19]. The loose facia between the prostate and the rectum was the Denonvillier’s facia in humans. To our knowledge, this structure has not been previously described in dogs. Schlake et al. [8] described a fascia similar to the fascia we observed. The Denonvilier’s facia is usually sutured to the posterior wall of the bladder before starting the VUA in human patients [16, 19]. This step was not required to bring the bladder closer to the urethra during our cadaveric study and was not performed during robotic prostatectomy in canine cadavers [9]. However, in human patients, suturing the fascia to the bladder might reduce the risk of extravasation of urine in the abdominal cavity in the postoperative period [20].

The completion of the VUA was the most challenging part of the procedure. Intracorporeal suturing is difficult during laparoscopy. After a radical prostatectomy, the VUA must be performed in the pelvic canal with limited space. The anastomosis is not performed in a very ergonomic situation since the suturing is performed with the end of the urethra facing the endoscope. The stitches have to be placed inside out in the urethra to avoid catching the other side of the urethra during suturing. The placement of stitches in the bladder was easier because the bladder wall was more mobile. It could then be placed in a more ergonomic position to place stitches. Using an endoscopic suturing device might facilitate intracorporeal suturing since it has been shown to improve suturing in difficult situations [21, 22]. Anastomosis is mainly performed with a continuous suture pattern in human patients [10–12, 23]. Eight to eleven stitches were placed to complete the VUA, similar to what has been described in the human literature [24]. The number of stitches placed with 2D and 3D cameras was not different. It is difficult to achieve a water-tight anastomosis in human patients since a urinary catheter and an abdominal drain are maintained from several days to weeks to limit the risk of uroabdomen while waiting for the VUA to seal [10, 25–31]. Special clips are commonly used in human patients to maintain tension on the continuous line of suture and provide a better seal [11, 24, 32]. Unidirectional barbed sutures are also used very frequently now because it allows for a better apposition of the tissue without maintaining tension like it is required with standard suture [27, 29, 33].

This study has several limitations. It was conducted in canine cadavers; therefore, hemostasis was not required. Hemostasis is an essential part of the procedure during transection of the urethra and dissection of the neurovascular pedicles. None of the canine cadavers had prostatic disease; all were intact male dogs. The presence of prostate neoplasia will make the dissection more challenging. Pressure testing of the VUA could not be conducted because of the nature of the frozen tissue. The suturing time was not reported because of the learning curve related to this procedure. We were more interested in accurate placement of the sutures with each camera than trying to make the suturing faster. Anatomical differences could have been significant confounding factors in comparing the suturing time between 2D and 3D groups. The endoscope/camera system we used for the study only has a 0-degree endoscope. A 30-degree endoscope may have helped during the dissection and the suturing.

In conclusion, laparoscopic radical prostatectomy has been described in canine cadavers and seems feasible in dogs with prostatic neoplasia. Experience with intracorporeal suturing is paramount. The 3-D camera may provide better depth perception for some parts of the dissection and the completion of the VUA.
